# Dura mater is a potential source of Aβ seeds

**DOI:** 10.1007/s00401-016-1565-x

**Published:** 2016-03-25

**Authors:** Gabor G. Kovacs, Mirjam I. Lutz, Gerda Ricken, Thomas Ströbel, Romana Höftberger, Matthias Preusser, Günther Regelsberger, Selma Hönigschnabl, Angelika Reiner, Peter Fischer, Herbert Budka, Johannes A. Hainfellner

**Affiliations:** Institute of Neurology, Medical University Vienna, AKH 4J, Währinger Gürtel 18-20, 1097 Vienna, Austria; Department of Medicine I and Comprehensive Cancer Center CNS Unit, Medical University Vienna, Vienna, Austria; Institute of Pathology, Danube Hospital Vienna, Vienna, Austria; Psychiatric Department, Medical Research Society Vienna, D.C., Danube Hospital, Vienna, Austria; Institute of Neuropathology, University Hospital Zurich, Zurich, Switzerland

**Keywords:** Alzheimer disease, Amyloid-β, Dura mater, Iatrogenic Creutzfeldt-Jakob disease, Prion, Propagon

## Abstract

Deposition of amyloid-β (Aβ) in the brain parenchyma and vessels is one of the hallmarks of Alzheimer disease (AD). Recent observations of Aβ deposition in iatrogenic Creutzfeldt-Jakob disease (iCJD) after dural grafting or treatment with pituitary extracts raised concerns whether Aβ is capable of transmitting disease as seen in prion diseases by the disease-associated prion protein. To address this issue, we re-sampled and re-evaluated archival material, including the grafted dura mater of two cases with iCJD (28 and 33-years-old) without mutations in the *AβPP*, *PSEN1* and *PSEN*2 genes, and carrying ε3/ε3 alleles of the *APOE* gene. In addition, we evaluated 84 dura mater samples obtained at autopsy (mean age 84.9 ± 0.3) in the community-based VITA study for the presence of Aβ deposition. We show that the dura mater may harbor Aβ deposits (13 %) in the form of cerebral amyloid angiopathy or amorphous aggregates. In both iCJD cases, the grafted dura mater had accumulated Aβ. The morphology and distribution pattern of cerebral Aβ deposition together with the lack of tau pathology distinguishes the Aβ proteinopathy in iCJD from AD, from that seen in young individuals without cognitive decline carrying one or two *APOE4* alleles, and from that related to traumatic brain injury. Our novel findings of Aβ deposits in the dura mater, including the grafted dura, and the distinct cerebral Aβ distribution in iCJD support the seeding properties of Aβ. However, in contrast to prion diseases, our study suggests that such Aβ seeding is unable to reproduce the full clinicopathological phenotype of AD.

## Introduction

Neurodegenerative diseases are characterized by progressive loss of neurons and dysfunction of functional systems associated with deposition of pathological forms of proteins predominantly in the central nervous system [31]. These proteins, such as amyloid-β (Aβ), tau, α-synuclein, TAR DNA-binding protein TDP-43 or prion protein (PrP) can be used as biomarkers. However, a further important aspect is whether these proteins are capable of transmitting disease between individuals, which would have significant public health implications. This issue has received support from experimental observations; furthermore, the hierarchical involvement of anatomical regions, considered as phases or stages of disease, has been also considered to support the prion-like spread of disease-associated proteins [9, 15, 21, 37, 55, 56]. Indeed, prion diseases are still the only protein misfolding disorders where human- or animal-to-human transmission has been proven. To fine-tune the terminology, recently the term propagon has been introduced and at least four conceptual levels have been defined, such as molecular, tissue, systemic and infectious propagons [15]. As suggested, only prion diseases fulfill all four levels, while this has not yet been shown convincingly for other neurodegeneration-related proteins [15, 37, 55]. However, recent reports showed that iatrogenic Creutzfeldt-Jakob disease (iCJD) due to cadaveric pituitary hormones [25] or dura mater implantation [18, 46] frequently associates with Aβ deposition, suggesting that Aβ acts as a seed or infectious propagon as defined recently [15]. However, these studies did not evaluate the original source material nor focused on distinctive neuropathological features of these cases.

Aβ deposition is one important component of neuropathological alterations that together with the accumulation of abnormally phosphorylated intracellular tau allows the tissue diagnosis of Alzheimer disease (AD) [14]. In contrast to prion diseases, AD is characterized by slowly progressive cognitive decline. Importantly, Aβ shows on one hand a maturation process [42] and on the other hand a hierarchical involvement of brain regions [53]. Moreover, two distinct patterns of neocortical involvement have been suggested, namely a homogenous distribution in all layers or a laminar pattern [12]. Cerebral amyloid angiopathy (CAA) with Aβ immunoreactivity is a frequent observation in the ageing and AD brains, which is thought to be related to failure of the interstitial fluid drainage of the brain [5, 10]. Two types of CAA are distinguished and three stages of brain involvement have been proposed [51, 52].

Importantly, Aβ plaques can be observed in young individuals associated with the presence of the ε4 allele of the apolipoprotein E (*APOE*) gene [45]. On the other hand neuronal phospho-tau pathology can be seen in subcortical nuclei from the second decade of life and precedes cortical involvement [7, 8, 20, 50]. Furthermore, accelerated neurodegeneration has been reported after traumatic brain injury (TBI) either after single or, as chronic traumatic encephalopathy (CTE), following repetitive brain trauma [27, 28, 40, 41]. Aβ deposits together with tau-positive neurofibrillary tangles reminiscent of AD are seen already after single TBI [28], while in CTE, tau pathology with stages of brain involvement is the major finding, which is associated with deposition of further neurodegeneration-related proteins, including Aβ [39–41].

In the present study we were thus interested to clarify whether (1) the pattern of Aβ deposition in iCJD following cadaveric dura mater implantation would be distinguishable from that seen in AD, in cognitively normal young individuals, or in TBI; (2) the dural grafts in iCJD cases would show Aβ deposition; and (3) the dura mater would accumulate Aβ in non-CJD cases. To answer these questions we re-sampled and carefully re-evaluated archival material, including the grafted dura mater of two cases with iCJD. In addition, we systematically evaluated dura mater samples collected in the community-based Vienna Trans-Danube Aging (VITA) study for the presence of Aβ deposition.

## Materials and methods

### Acquisition of cases

Two cohorts were examined in this study. The first cohort consisted of two autopsy cases of iCJD collected in the frame of the Austrian Surveillance for human prion diseases. Both patients underwent dura mater transplantation: case iCJD-1 was a 33-year-old female patient, and iCJD-2 was a 28-year-old male patient. The latter case was reported previously [46]. We re-evaluated both cases carefully and sampled further anatomical areas including the implanted and host dura mater. The second cohort consisted of 84 consecutive dura mater samples collected in the frame of the community-based VITA study [16, 34]. The mean age at death (±standard error) of individuals in the VITA cohort was 84.9 ± 0.3 (range 79–89 years); male/female ratio was 33/51). The study was performed following local regulations as approved by the local ethical committee (EK07-056-VK, 206/05/2008, EK396-2011).

### Neuropathology

Formalin-fixed, paraffin-embedded tissue blocks were evaluated. Tissue blocks examined in iCJD cases comprised bilateral samples of the frontal (frontobasal area of traumatic surgery and dorsolateral), anterior cingular, lower, middle and upper temporal, parietal, and occipital cortices with white matter, hippocampus, entorhinal cortex, basal ganglia, thalamus, mesencephalon, pons, medulla oblongata, and cerebellum. In case iCJD-1 we evaluated the implanted and immediately adjacent host dura mater. In case iCJD-2 the whole dura mater was available; thus we evaluated samples from the implanted areas and from those parts of the dura mater not involved in the traumatic injury (host dura, at least 5 cm distance from the surgical intervention from both sides).

In the VITA cohort we sampled the left temporal region of the dura mater including branches of the middle meningeal artery (2 × 2 cm) as well as three cross-sections in the area of the superior sagittal sinus and confluence of sinuses. In addition to Hematoxylin and Eosin (HE), Congo red, van Gieson elastica, and Bielschowsky stainings, the following monoclonal antibodies were used for immunohistochemistry: anti-PrP (1:1000, 12F10, Cayman Chemical, Ann Arbor, MI, USA), anti-Aβ (1:100, clone 6F/3D, Dako, Glostrup, Denmark), anti-Aβ (1:4000, clone 4G8, Signet, San Diego, CA, USA), anti-Aβ_1-40_ (1:800, clone 139-5, Signet, San Diego, CA USA), anti-Aβ_1–42_ (1:200, clone 1-11-3, Signet, San Diego, CA USA), anti-AβPP (1:8000, clone 22C11, Millipore, Temecula, CA, USA), anti-phospho-tau (1:200, clone AT8, Pierce Endogen, Waltham, MA, USA), smooth muscle actin (1:200, clone HHF35, Dako, Glostrup, Denmark), anti-ubiquitin (1:50,000, Millipore, Temecula, CA, USA), anti-HLA-DR (1:100, clone CR3/43, Dako), anti-CD-68 (1:5000, clone KP1, Dako), and collagen IV (1:100, clone CIV22, Dako, Glostrup, Denmark; to label basement membrane). The following tissue pretreatments were used for the Aβ antibodies prior to incubation with primary antibodies: for 6F/3D and 4G8 1 h 80 % formic acid (FA); for anti-Aβ_1–40_ and anti-Aβ_1–42_ 10 min 70 % FA, for AT8 no pretreatment. Additional enhanced Proteinase K (PK) treatment (50 μg/ml) was used for 6F/3D and 4G8 for 5 min at 37°C to test for PK resistance of the detected dural Aβ deposits. The Dako EnVision™ FLEX + Mouse / Rabbit detection system (Dako, Glostrup, Denmark) was used for visualization of antibody reactions.

In the VITA cohort we correlated the presence of different Aβ deposits in the dura with neuropathological variables, including presence and phase of Aβ deposition, presence and type of CAA, stages of neurofibrillary degeneration, presence of Lewy body, TDP-43 and vascular pathology (see Ref. [34]). Chi square test and Fisher’s exact test were used to evaluate association between variables of interest. IBM SPSS Statistics Version 20 was used for statistical analysis. A significance level of 0.05 was used.

### Gene analysis

Genomic DNA was isolated using the QIAamp DNA Mini Kit (QIAGEN, Hilden, Germany) according to the manufacturer’s instructions using frozen brain tissue of the two iCJD cases (Case 1 and 2). All primer pairs were designed to amplify the coding exons of *PSEN1*, *PSEN2* (presenilin 1, 2), *PRNP* (prion protein gene) and exon 16 and 17 of *AβPP* (Aβ precursor protein) including adjacent exon/intron boundaries. The PCR fragments were purified by agarose gel electrophoresis and sequenced using the Dye Terminator cycle sequencing kit (Version 3.1; Applied Biosystems, Foster City, CA) with electrophoresis on an ABI 3130 Genetic Analyzer (Applied Biosystems). To genotype *APOE*, restriction fragment length polymorphism was used as described previously [22]. In brief, a fragment within exon 4 of the *APOE* gene was amplified by PCR. Then the amplified 244 bp fragment was cut with restriction enzyme HhaI. Restriction fragments were separated on a 16 % polyacrylamide gel and visualized.

## Results

### Description of cases examined

Case iCJD-1 was a 33-year-old woman who had suffered a traumatic open right frontobasal skull fracture followed by surgical implantation of lyophilized cadaveric dura 25 years before death. After the operation epileptic seizures occurred; the last one 15 years before death. She developed progressive dementia at the age of 33 associated with cerebellar ataxia, myoclonus and pyramidal signs. She died due to bronchopneumonia after 4 months disease duration. CSF 14-3-3 protein was positive and EEG revealed triphasic waves. There was a lack of reports on amnestic or focal cortical symptoms before the development of progressive neurological symptoms. There was no family history of dementia or any other neurological disease.

Case iCJD-2 was a 28-year-old man who had suffered a traumatic open left frontobasal skull fracture with brain contusion and tearing of the dura mater 22 years before death [46]. The dural defect was surgically covered using lyophilized cadaveric dura. At the age of 27 he developed progressive dementia, myoclonus and seizures, later pyramidal signs and akinetic mutism. There were no preceding symptoms. CSF 14-3-3 protein was positive, EEG revealed periodic sharp waves, and cranial MRI showed discrete hyperintensity of the caudate nucleus and putamen in T2 weighted images. Two months before death a diagnostic brain biopsy of the left frontal lobe was performed. Disease duration was 8 months. There was no family history of dementia or any other neurological disease.

Genetic analysis of presenilin 2 (*PSEN2*) revealed polymorphic sites at codons 23, 43 and 87 (c.69T>C; p.Ala23Ala, c.129C>T; p.Asn43Asn and c.261C>T; p.His87His, respectively) in both iCJD patients. These polymorphisms have been reported as having no pathogenic impact. In *AβPP* exons 16 and 17, presenilin 1 (*PSEN1*) and *PRNP* no pathogenic mutation was observed in both patients. Case iCJD-1 was heterozygous (methionine/valine) and iCJD-2 was homozygous for methionine at the polymorphic codon 129 of *PRNP*. Both cases carried only ε3 alleles (ε3/ε3) in the *APOE* gene.

### Neuropathology of iCJD cases

**Fig. 1:**
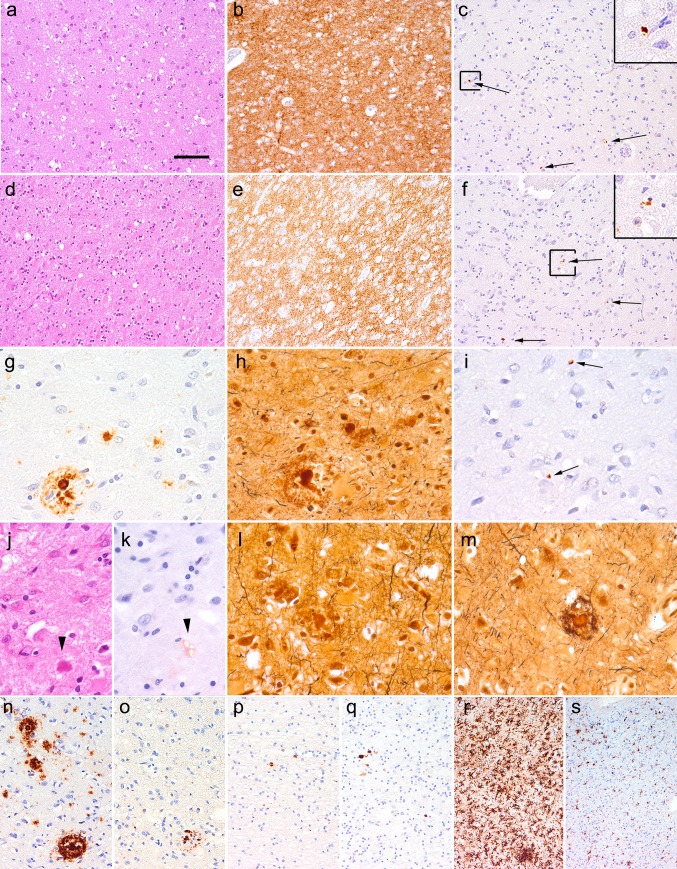
Neuropathology of iCJD cases. Mild to moderate spongiform change in the HE staining (**a**, **d**) associated with diffuse/synaptic PrP immunoreactivity (**b**, **e**) and focal tau immunoreactive neuritic profiles (**c**, **f** indicated by *arrows*, a representative one is enlarged in *right upper inset*) in case iCJD-1 (**a**–**c**) and iCJD-2 (**d**–**f**). The same mature plaque with a corona in iCJD-2 as seen in immunostaining for Aβ (**g**), Bielschowsky (**h**), AT8 (**i** *arrows* indicate small neuritic profiles as seen in CJD cases but not around the mature plaque), HE (**j** *arrowhead*) and Congo staining (**k** *arrowhead* indicates the plaque as seen under polarized light). Bielschowsky staining (**l**, **m**) of two mature plaques lacking tau immunoreactivity close to each other in iCJD-2. Immunostaining for Aβ (**n**) and ubiquitin (**o**) in the same cortical regions close to the dura transplant in iCJD-1. Immunostaining for AβPP in frontal white matter in iCJD-1 (**p**) and iCJD-2 (**q**). Immunostaining for HLA-DR (microgliosis) in the frontal cortex (**r**) and white matter (**s**) in iCJD-1. The *bar* in image “**a**” represents 100 μm for **a**–**f**, **r**, **s**; 40 μm for **g**–**m**; and 60 μm for **n**–**q**

Histology of both iCJD cases revealed mild to moderate spongiform change associated with prominent gliosis and neuronal loss and diffuse/synaptic immunoreactivity for disease-associated PrP (Fig. 1a–f). There were no PrP plaques or kuru type plaques. The pattern was compatible with MV/MM type 1 [44]. Immunostaining for Aβ revealed parenchymal plaques and CAA in both cases (see below). Immunostaining for phosphorylated tau (AT8) revealed occasional small neuritic profiles as described in different forms of CJD [32, 47]. However, there was a complete lack of neurofibrillary tangles, pretangles (including subcortical nuclei), neuropil threads, or glial tau immunoreactivity. We did not observe any tau immunoreactive dystrophic neurites surrounding Aβ plaques although Bielschowsky silver staining did visualize the mature and immature plaques, but mostly without abundant argyrophilic neuritic components (Fig. 1g–i). In the HE staining we observed several amyloid cores in both cases, showing also birefringence under polarized light in the Congo red staining (Fig. 1j, k). Only very rare mature plaques showed weakly stained argyrophilic neurites (Fig. 1l, m), which were however AT8 negative. Immunostaining for ubiquitin revealed neuritic profiles around some mature plaques (Fig. 1n, o). In the white matter close to the area of the traumatic lesion focal AβPP deposits were seen in both cases (Fig. 1p, q). In case iCJD-2 there was a mononuclear granuloma-like inflammation in the area of the brain biopsy in the left frontal lobe together with ventriculitis and meningitis with mononuclear inflammatory infiltrates, interpreted as inflammatory sequel of CSF leakage following the biopsy [46]. Microglial reaction, together with macrophages, was most prominent in the cortex and white matter close to the lesion (Fig. 1r, s), while in other areas mostly the cortex showed moderate accumulation of ramified microglia. In iCJD-2 microgliosis was much more prominent due to the recent inflammation.

### Distribution and morphology of dural and brain Aβ deposits in the iCJD cases

Both cases showed similar morphology of Aβ immunoreactivities, characterized by CAA (type 2) [51] and predominantly focal cortical deposits, including cored mature plaques, immature primitive plaques and focal cores without a surrounding corona. Parenchymal Aβ deposits accumulated closer to the area of the operation (Table 1), appeared in clusters, and were seen mostly in the frontal and temporal cortex. In iCJD-2 we observed mature and primitive plaques and focal deposits in the anterior cingulate gyrus on the lesion side; furthermore, occasional perivascular focal deposits in the occipital cortex and occasional plaques in the parietal cortex were also observed. Accordingly, both cases were classified as phase 1 according to Thal et al. [53], with the note that in iCJD-2 the involvement of the anterior cingulate suggested phase 2, however, the complete lack of parenchymal Aβ deposits in the entorhinal cortex, hippocampus, and insular cortex deviated from the established phase 2. CAA was seen in neocortical areas but not in subcortical areas or cerebellum (stage 1) [52]. There was a lack of dyshoric angiopathy or fibrinoid necrosis of vessels. In case iCJD-1 mostly the leptomeningeal vessels showed CAA, while in iCJD-2 the cortical vasculature additionally exhibited prominent CAA.

**Table 1:** Anatomical distribution of Aβ deposits in the two cases of iatrogenic Creutzfeldt-Jakob disease (iCJD). Focal Aβ deposits are stratified as mature (classic) plaques and other (immature primitive plaques or compact plaques). Diffuse Aβ deposits include here only subpial deposits. If there was no difference between the two sides (R: right, L: left) they are summarized in one row. Further Aβ deposition indicates lake-like immunoreactivity in the lesion area, deposits in a venous dilatation (temporal in iCJD-1) and amorphous deposits in the dura

Region/Aβ	iCJD-1					iCJD-2				
	CAA	Focal		Diffuse	Further	CAA	Focal		Diffuse	Further
		Mature	Other				Mature	Other		
Frontobasal L^a^	−	−	+	−	−	++	++	++	+	−
Frontobasal R^b^	++	+++	+++	−	+++	+	−	+	−	+
Middle Fr L	−	+	+	−	−	++	++	++	−	−
Middle Fr R	+	−	+	−	−	+	−	+	−	−
Middle-Upper Te L	+	+	+	−	−	+	−	++	+	−
Middle-Upper Te R	+	+	+	−	+	+	−	+	−	−
Inferior Pa R-L	−	−	−	−	−	+	+	+	−	−
Occ R-L	+	−	−	−	−	+	−	+	−	−
Hippocampus^c^ R-L	−	−	−	−	−	+	−	−	−	−
Basal GGl R-L	−	−	−	−	−	−	−	−	−	−
Thalamus R-L	−	−	−	−	−	−	−	−	−	−
Mesencephalon	−	−	−	−	−	−	−	−	−	−
Pons	−	−	−	−	−	−	−	−	−	−
Medulla oblongata	−	−	−	−	−	−	−	−	−	−
Cerebellum	−	−	−	−	−	−	−	−	−	−
Dura graft	+	−	−	−	+	+	−	−	−	+
Dura host^d^	−	−	−	−	−	−	−	−	−	−

*R* right, *L* left, *Fr* frontal, *Te* temporal, *Pa* parietal, *Occ* occipital, *GGl* Ganglia, − indicates negative, +, ++, +++ indicates positive (mild, moderate, severe, respectively)

^a^Site of dural grafting in iCD-2

^b^Site of dural grafting in iCD-1

^c^Cornu ammonis, subiculum, entorhinal and inferior temporal cortex

^d^Host dura was examined beside the grafted dura in iCJD-1 and more than 5 cm away from the operation site in iCJD-2

**Fig. 2:**
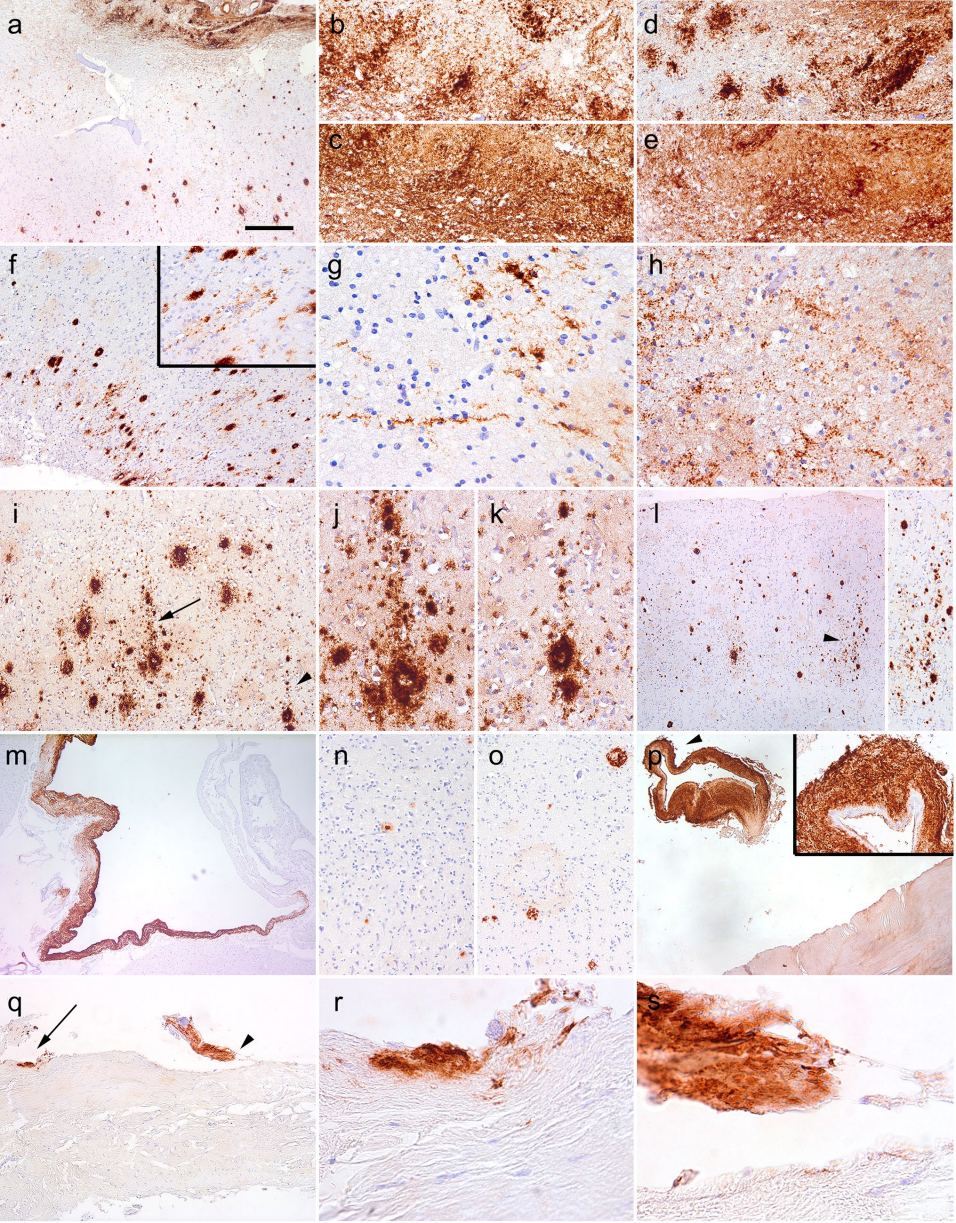
Aβ immunoreactivity in iCJD-1. Immunostaining using the 6F/3D Aβ antibody (**a**, **b**, **f**, **g**, **i**–**m**, **p**–**s**), the 4G8 Aβ antibody (**c**, **h**), anti-Aβ_1–40_ (**e**, **n**), and anti-Aβ_1–42_ (**d**, **o**) demonstrated widespread immunoreactivity in the lesion area (**a** *upper part* enlarged in **b**–**e**); radiating deposits in the cortex (**f**) and white matter (**g**, **h**); focal deposits with columnar alignment perpendicular to the surface of the cortex (**i**–**l**); in a dilated vein (**m**), as immunostaining of plaques (**n**, **o**); and as dural deposits (**p**–**s**). The *right upper inset* in **p** is the enlargement of the area indicated by an *arrowhead*; **r** and **s** are enlargements of areas indicated by *arrow* and *arrowhead* in **q**, respectively. The *bar* in image “**a**” represents 150 μm for **a**, **f**, **l**, **m**–**o**; 60 μm for **b**–**e**; 40 μm for **g**, **h**, **j**, **k**; 100 μm for **i**, **p**, **q** and 15 μm for **r**, **s**

In case iCJD-1, the traumatic lesion area where the graft was implanted showed prominent tissue damage. Aβ showed unusual lake-like appearance in the white matter adjacent to the tissue defect, strongly labeled by Aβ_1–40_ and Aβ_1–42_ as well (Fig. 2a–e). Aβ deposits radiated from the lesion (Fig. 2f) and included linear and fine granular, but not axonal bulb-like Aβ depositions in the perilesional white matter (Fig. 2g, h) and at the gray-white matter junction. In addition, in the adjacent cortical area, columns of Aβ deposits, oriented perpendicular to the brain surface, were noted (Fig. 2i–l). In further cortical areas, with decreasing density away from the lesion (Table 1), either single cores or clusters of plaques, both primitive and mature, were seen. CAA involved mostly the meningeal vessels including large venous dilatations (Fig. 2m) and the cortical arteries were less involved. The plaques and CAA were observed using all anti-Aβ antibodies; Aβ_1–40_ showed mostly CAA and the cores of plaques, while Aβ_1–42_ immunolabeled the corona of the plaques as well (Fig. 2n, o). The grafted dura mater also showed amorphous Aβ depositions including adjacent sinus-like dilatations (Fig. 2p–s).

**Fig. 3:**
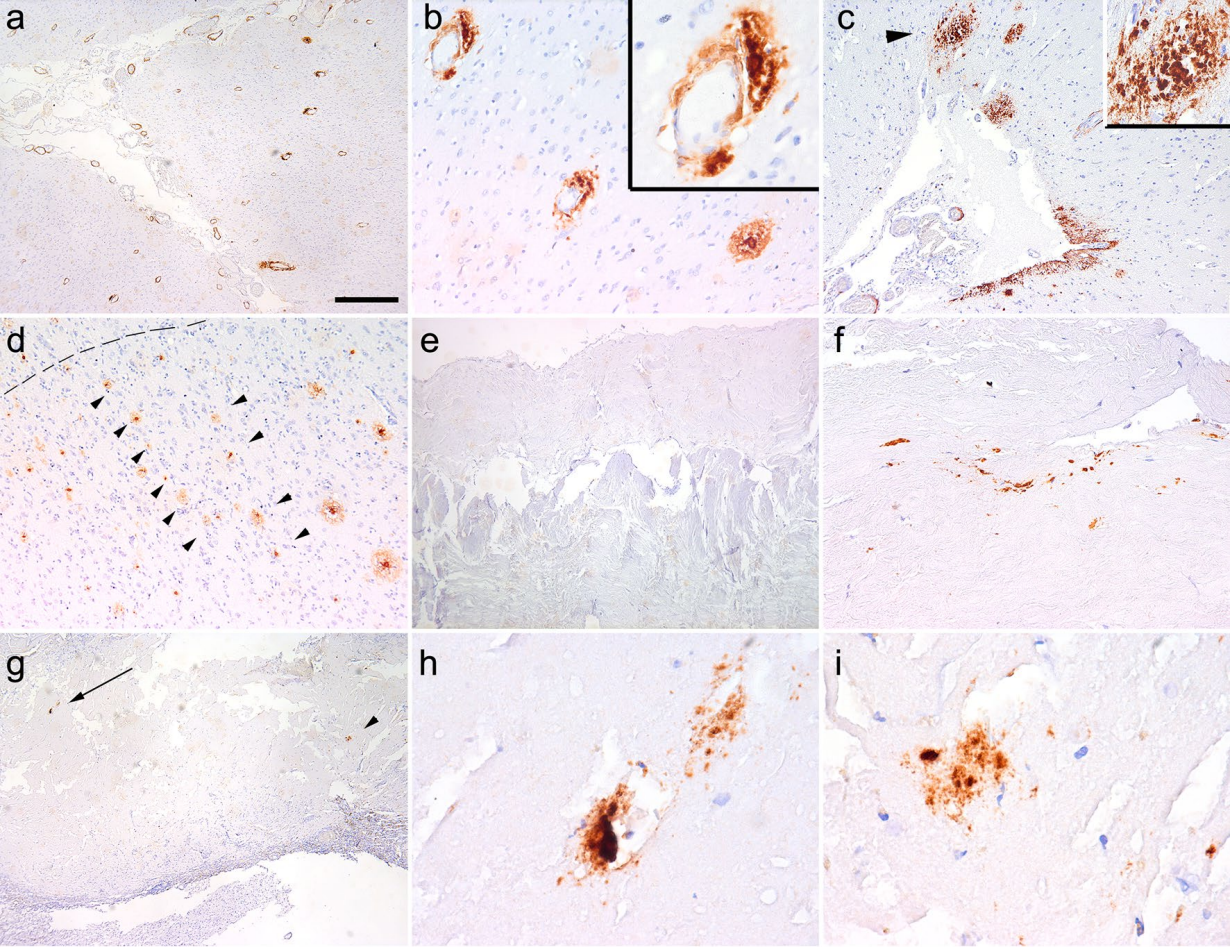
Aβ immunoreactivity in iCJD-2. Immunostaining using the 6F/3D Aβ antibody (**a**–**i**) representing cerebral amyloid angiopathy (**a**), perivascular cored plaque (**b** *upper right inset* is the enlarged image of the vessel below the letter “**b**”), single area with subpial deposits (**c** including minicored plaques indicated by an *arrowhead* and as enlarged in the *right upper inset*); columnar alignment of plaques in the cortex (**d** indicated by a row of *arrowheads*; the surface of the cortex is labeled by a *dashed line*); lack of immunoreactivity in the host dura mater (**e**) and amorphous Aβ depositions in the graft dura mater (**f**–**i**; **h** and **i** is the enlargement of the areas indicated by an *arrow* and *arrowhead* in **g**, respectively). The *bar* in image “**a**” represents 150 μm for **a**; 40 μm for **b**, **f**; 100 μm for **c**, **d**, **e**, g; 15 μm for **h**, **i**

In case iCJD-2 we observed two focal deposits and a single vessel with CAA already in the brain biopsy from the frontal cortex. In the post mortem specimens the operated area showed prominent inflammatory changes. In addition to CAA (Fig. 3a), we observed perivascular focal deposits, including cored plaques (Fig. 3b). Subpial diffuse deposits were seen only focally in the temporal lobe (Table 1), and these also showed mini-cores (Fig. 3c). Furthermore, cortical Aβ deposition showed clustering of plaques, which were mostly focal deposits including mature and primitive plaques (Fig. 3d). In preserved cortical areas adjacent to the lesion we recognized columns of Aβ deposits perpendicular to the surface (Fig. 3d). Several samples of dura mater from the host (more than 5 cm from the operated area) lacked Aβ deposition (Fig. 3e). In contrast, there was amorphous Aβ immunoreactivity in the grafted dura (Fig. 3f–i).

In summary, common features comprise (1) amorphous Aβ deposits in the grafted but not host dura mater; (2) presence of parenchymal deposits (iCJD-1 > iCJD-2) and CAA (iCJD-2 > iCJD-1); (3) predominance of focal deposits (mature and immature plaques) and lack of ill-defined irregular diffuse plaques; (4) predominance of plaques in areas close to the traumatic lesion where the graft was implanted; (5) clustering of plaques in cortical areas without laminar preference or homogenous involvement of all layers; (6) tendency for columnar alignment of focal deposits perpendicular to the surface in the vicinity of the lesion; and (7) complete lack of neuronal or glial tau pathology and particularly of plaque-associated tau-positive dystrophic neurites.

### Morphology and frequency of dural Aβ deposits in the VITA cohort

**Fig. 4:**
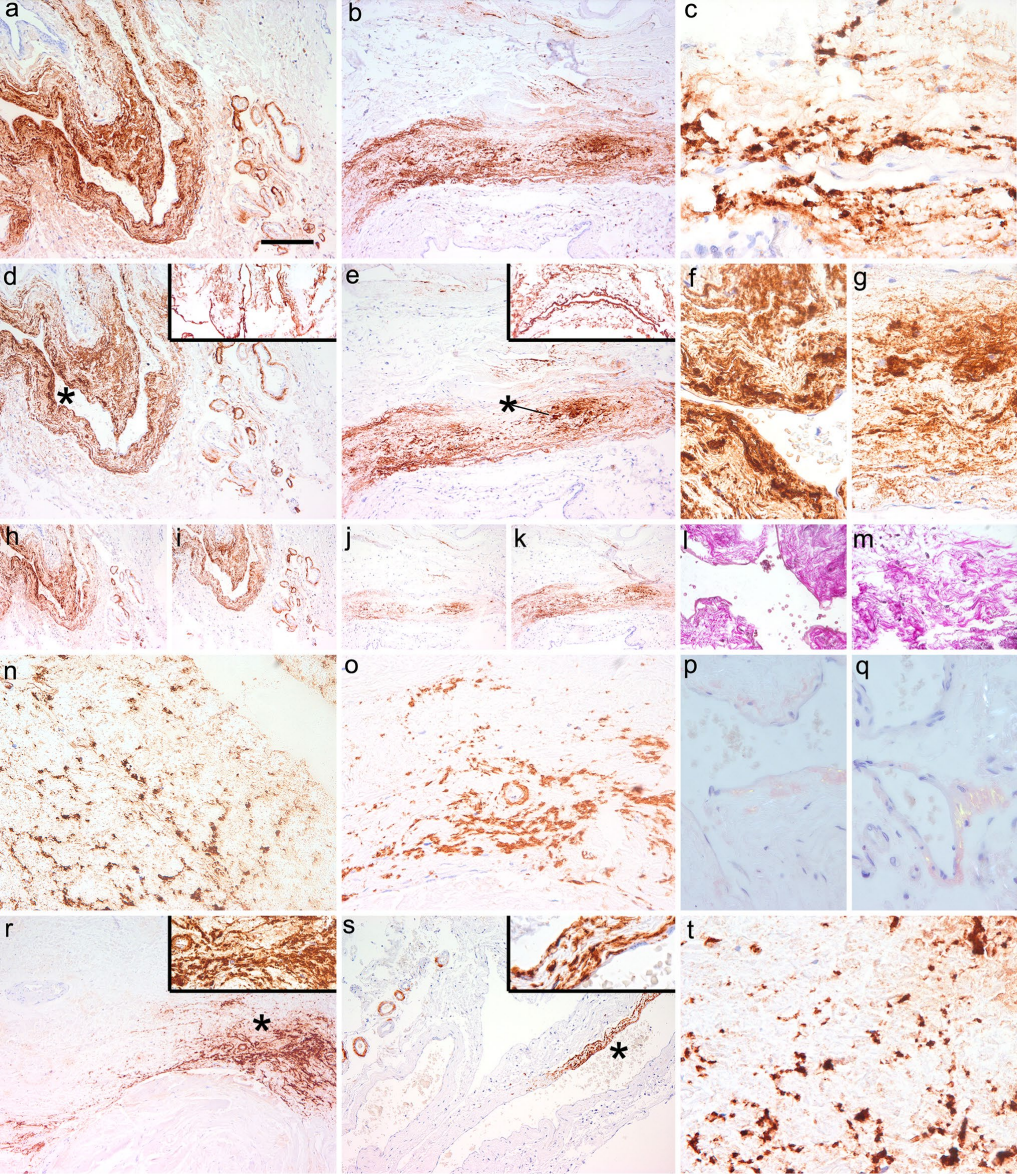
Aβ in the dura mater in the aging brain. Image **a**–**m** represents dura mater samples from a representative patient as visualized by 4G8 (**a**–**c**; in **c** using enhanced PK treatment of the sections), 6F/3D Aβ antibody (**d**–**g**; *insets* in **d** and **e** represent immunostaining for collagen IV; **f** and **g** are enlargements of the areas indicated by *asterisk* in **d** and **e**, respectively), Aβ_1–40_ (**h**, **j**), Aβ_1–42_ (**i**, **k**) and van Gieson elastica (**l**, **m**). Further representative cases are shown using immunostaining for Aβ_1–40_ (**n**), Aβ_1–42_ (**o**), Congo staining under polarized light (**p** deposit in the connective tissue, **q** angiopathy), and 6F/3D (**r**–**t**; areas indicated with *asterisk* enlarged in the *right upper inset*; in **t** using enhanced PK-pretreatment). The *bar* in image “**a**” represents 20 μm for **c**, **f**, **g**, **n**, **p**, **q**, **t**; 40 μm for **a**, **b**, **d**, **e**, **o**, **r**, and **s**; and 80 μm for **h**–**m**

In the VITA cohort we observed typical morphology of CAA with congophilia in 11/84 cases (13.09 %; median age: 85 years, range 82–89); and amorphous deposits of Aβ in the connective tissue mostly adjacent to dural sinuses in 11/84 cases (13.09 %; median age: 86 years, range 84–88; only five of these cases with CAA also). The age was not significantly different between groups showing or lacking Aβ immunoreactivity in the dura. Both CAA and the amorphous deposits were detectable by the 4G8, 6F/3D, anti-Aβ_1–40_, and anti-Aβ_1–42_ antibodies (Fig. 4a–o, r, s). In the connective tissue, where we observed amorphous Aβ immunoreactivity, collagen IV decorated basement membranes (Fig. 4d, e inset). Combined presence of CAA and amorphous deposits was seen only in five cases. Using enhanced proteinase K treatment, both types of lesions were detectable by both 4G8 and 6F/D3 anti-Aβ antibodies (Fig. 4c, t); furthermore, focally they showed birefringence in Congo staining under polarized light (Fig. 4p, q). In addition, some vessels showed fine granular deposits without congophilia, detectable only by the 4G8 antibody in the media of arteries, in 41/84 cases (48.8 %; median age: 85 years, range 82–89). Detectability of these morphologies using anti-Aβ antibodies did not correlate with the duration of the formalin fixation of samples.

Next we compared the presence of the different morphologies with neuropathological variables including the presence of Lewy body pathology, TDP-43 proteinopathy, tauopathy, parenchymal Aβ deposits or CAA in the brain or leptomeningeal vessels, vascular lesions in the brain and Braak neurofibrillary stage. It is of note that cases with the presence of typical CAA detected by all antibodies in the examined dura samples showed Aβ deposits in the brain (100 %), while 8 (72.7 %) of them showed also CAA in the examined brain samples. Similarly, dural amorphous deposits were significantly associated with Aβ deposits in the brain (100 %), and in 10 cases (90.9 %) there was also CAA in the brain and leptomeningeal vessels (*p* < 0.01 according to *χ*^2^ and Fisher exact test for both comparisons). In contrast, presence of fine granular Aβ immunoreactivity detectable only with the 4G8 antibody in the media of large arteries did not show correlation with any of these. Furthermore, none of the dural Aβ morphologies was associated with tau, TDP-43, Lewy body, or vascular pathologies detected in the brain.

## Discussion

To contribute to the emerging issue of potential transmissibility of neurodegenerative conditions and related proteins we re-evaluated two iCJD cases in which the grafted dura mater was available. In addition, we addressed the question whether Aβ can deposit in the dura mater in aged individuals.

### Comparison of Aβ pathology with sporadic AD and TBI-related neurodegeneration

In sporadic AD, deposits of Aβ are classified as diffuse, stellate and focal [14]. Diffuse deposits are poorly immunoreactive and their borders are ill-defined. Depending on the localization they show lake-like or fleecy morphology or they appear as subpial bands. Focal deposits are different and they are further stratified whether they have a neuritic corona or not [2, 14]. Importantly, a grading system was suggested for these lesions, beginning with purely focal Aβ deposition, followed by appearance of Congo red positive material with neurites and variable degree of ubiquitination, which later show immunoreactivity for hyperphosphorylated tau, and finally associate with neurofibrillary tangles in the vicinity of plaques [42]. Importantly, diffuse Aβ deposits are considered as the earliest form, while mature plaques can be seen later [14, 15]. Another important aspect of Aβ pathology is the hierarchical and stereotypical involvement of different brain regions, which follows five distinct phases [53]; the neocortex is involved in the first phase. Moreover, the study by Cupidi et al. suggested considerable heterogeneity of neocortical Aβ deposition in late phases of AD [12]. That study identified two patterns: a laminar intracortical pattern and another one where the six isocortical layers were homogeneously involved [12]. Further studies showed that diffuse and focal deposits distinctly appear in different layers of the cortex [13], which was not seen in our cases. Indeed, the two iCJD cases examined here revealed a distinct pattern. On one hand there was neither an unequivocal laminar pattern nor homogenous involvement of cortical layers even in regions with many mature and primitive plaques. Aβ deposition frequently appeared in clusters away from the lesion site or showed columnar alignment close to the lesion. On the other hand, the maturation process of plaques [42] was not recognizable; neither diffuse Aβ deposits with ill-defined borders, nor any tau immunoreactivities in the corona of mature plaques were seen. Thus, in spite of the presence of mature plaques, only grade 2 (out of 4 as proposed by Metsaars et al [42].) of isocortical Alzheimer lesions could be recognized. Therefore, we conclude that the Aβ deposition seen in our iCJD cases is distinct from that seen in AD. It must be noted that Αβ immunoreactivity frequently appears in certain forms of genetic CJD (e.g. E200K mutation) even in younger patients [19, 35]; furthermore, young individuals without cognitive decline may have Αβ deposits in the neocortex [45]. Importantly, these are also different from that seen in the present iCJD cases, since they are predominated by irregular diffuse plaques with ill-defined border lacking amyloid cores. Moreover, in young individuals the presence of the ε4 allele of the *APOE* gene is significantly associated with the appearance of Aβ plaques [44]. Our iCJD cases, representing the youngest cases where Aβ deposits were observed in iCJD [18, 25], carried only ε3 allele in the *APOE* gene; furthermore, there was a lack of pathogenic mutations in genes associated with altered Aβ metabolism (i.e. *AβPP*, *PSEN1, PSEN2*). It is important to emphasize that apart from the sparse small neuritic profiles detected in all prion disease types [32, 35, 47], we did not observe neuronal tau pathology in subcortical neuronal groups [7, 8, 20, 50] or thread like profiles [33] as reported in young individuals.

A possible explanation for the increased frequency of Aβ pathology in our iCJD cases might be simply the effect of brain trauma. Recent studies indicate that following repetitive injury CTE develops, which pathognomistically shows tau pathology [39], although Aβ deposition can be additionally seen [40, 41]. In our patients there was neither repeated trauma documented nor were any clinical symptoms or tau pathologies compatible with CTE observed. Another form of neurodegeneration has been documented in individuals many years after a single TBI, showing Aβ but also tau deposition [28]. We also observed chronic lesions, such as accentuated microgliosis and macrophages and occasional axonal bulbs, which may persist years after TBI as described by Johnson and colleagues [26]. In contrast, TBI cases were reportedly less likely to display smaller clustered regions of plaques and more likely to have widespread plaques across the entire cortex [28]. Together with the lack of tau pathology in the iCJD cases examined, the distribution of plaques has to be emphasized: it showed higher density close to the dura mater graft. Interestingly, another study on TBI did not find Aβ plaques in long-term survivors suggesting regression with time [11]. In short-term survivors of acute TBI mostly Aβ_1–42_ immunoreactivity was seen as non-neuritic plaques [23]. A recent study using the amyloid tracer ^11^C-Pittsburgh compound B to evaluate amyloid pathology in vivo in individuals with a history of TBI (11 months to 17 years) demonstrated a distinct involvement of the cerebellum [49] unlike in our patients. Importantly, the images showed no binding in the vicinity of focal cortical lesions in TBI evident on structural MRI [49], which also contrasts with our iCJD cases where most of the amyloid pathology was in cortical regions close to the graft. Finally, ε4 carriers of the *APOE* gene have been discussed to be at increased risk for developing Aβ pathology after TBI [27].

Interestingly, CAA is less emphasized in TBI. Less than 10 % frequency has been reported in individuals with TBI shortly after the injury, mostly associated with the presence of the *APOE* ε4 allele [36]. CAA was seen in the brain in ex-boxers [54]. In sporadic CAA the pathogenesis includes altered clearance or increased production of Aβ [52]. In the present iCJD cases CAA was observed only in the leptomeninges and in the neocortex (stage 1). It might be theorized that the grafted dura containing Aβ deposits impaired the physiological clearance facilitated by additional seeding of Aβ to the vessels. Interestingly, perivascular Aβ deposits frequently showed cores (Fig. 3b), which is unusual in AD.

In summary, the pathology seen in the two iCJD cases was distinct not only from AD brains but also from that described in long-term survivors of TBI or in CTE. The observation of Aβ deposits in the grafted but not in the host dura mater suggests a scenario of seeding of the pathological protein to the underlying CNS. However, the Aβ seeds alone seem to be inefficient in reproducing the complete clinicopathological phenotype of AD. Lack of the clinical phenotype of AD has been emphasized in recipients of cadaveric human growth hormone [24]. This contrasts with prion diseases, where the seeding of disease-associated PrP leads to widespread involvement of the brain together with the entire phenotype.

### Dural Aβ pathology in the aging brain

Next we addressed the question how frequently and in what form does Aβ pathology involve the dura mater in aging. In spite the small size of the dura sample examined (4 cm^2^) we were able to detect deposits associated with AD type pathology in the CNS. CAA and amorphous deposits were labeled by all antibodies even following harsh PK-pretreatment of sections and showed birefringence in Congo red staining using polarized light. These morphologies were similar to that seen in the grafted dura samples in iCJD cases. The fine granular immunoreactivity detectable only with antibody 4G8 in the media of vessels showed no specific correlation with neuropathological variables and was not interpreted as CAA, in particular since antibody 4G8 also detects intracellular AβPP [1].

It was suggested that amyloid proteins have a ubiquitous affinity to basement membranes [6] as demonstrated for dura-related Aβ deposits in our study and also by Keable and colleagues [29]. Indeed, amyloid deposits in the dura mater (pachymeninx), but not in the leptomeninges, have been shown in cases with generalized (systemic) amyloidosis, which on the other hand involve CNS tissue usually only in regions where the blood brain barrier is not sufficient [6]. In contrast, cerebral Aβ amyloidosis predominantly affects CNS tissue and vessels in the CNS and leptomeninges [6]. Here we expand the spectrum of tissues showing Aβ deposition in cerebral Aβ amyloidosis by demonstrating that the dura mater indeed accumulates pathological Aβ without selectivity for Aβ_1–40_ or Aβ_1–42_ in the amorphous deposits. It has long been suggested that the dura mater is a metabolically inert, avascular, fibrous covering of the brain. However, the dura mater also contains basal membranes [3], including in the abundant microvascularization, which is not commensurate with the role previously attributed to the dura mater [48]. Many studies have suggested that CAA is a protein elimination failure angiopathy where CAA reflects impaired perivascular lymphatic drainage (for reviews see [5, 10, 57]). Until recently, however, the lymphatic system of the dura mater received less attention. Early studies already emphasized that the lymphatic system might play a role in the fluid circulation of the brain [17, 30]. Interestingly, recent studies confirmed that a lymphatic system lining the dural sinuses drains the brain interstitial fluid [4, 38], which might have relevance to understand our observation of the accumulation of amorphous Aβ deposits in the vicinity of dural sinuses in a cohort of elderly individuals.

## Conclusions

We provide novel observations complementing a recent study on the increased frequency of Aβ pathology in iCJD associated with dura mater transplantation [18]. The pattern of Aβ deposition together with the lack of accompanying tau pathology differentiates the Aβ proteinopathy in iCJD from sporadic and genetic forms of AD; from that seen in young individuals without cognitive decline carrying one or two *APOE4* alleles; from that in young genetic CJD patients with mutation in the *PRNP* gene; and from that related to TBI and CTE. Additionally, the presence of Aβ in the grafted but not the host dura mater suggests that we observe here an example of ‘infectious propagon’ [15]. Infectious propagons have been defined as “proteins that transmit pathological conformation between individuals” [15]. However, of significance is the lack of clinical symptoms reminiscent of AD and of the full neuropathological picture of AD, which contrast with the phenotype-reproducing property of prions in prion diseases. In fact, we demonstrate here that pathological Aβ protein, rather than AD, can be propagated between humans. Therefore, we propose to distinguish Aβ as an ‘infectious propagon’ (when only the pathological conformation is transmitted between individuals) from disease-associated PrP as a ‘phenotype propagon’ (when the pathological conformation of the protein and the full clinicopathological phenotype is transmitted between individuals; e.g. prion disease). This concept would also help to better understand reports on horizontal transmission of other amyloids like the AA amyloid in captive cheetahs [43]. In addition, our observations in a cohort of elderly individuals show that the dura mater does not infrequently harbor Aβ, especially in the vicinity of sinuses, which provides an important aspect to the understanding of the drainage of this protein from the CNS.
